# Effects of social support interventions on depressive symptoms and quality of life among older adults: a systematic review and meta-analysis

**DOI:** 10.1186/s12877-025-06146-7

**Published:** 2025-07-09

**Authors:** Joel O. Faronbi, Blessing Eromosele, Henrietta O. Fawole, Opeyemi A. Idowu, Olayinka Akinrolie, Adesanmi Akinsulore, Tolulope Adeniji, Michael Chigozie Ibekaku, Oluwagbemiga Oyinlola, Grace O. Faronbi, Chidozie Mbada

**Affiliations:** 1 Academy of Nursing, Department of Health and Care Professions, University of Exeter, Exeter, Great Britain; 2 Department of Physiotherapy, University of Benin, Benin-City, Edo State Nigeria; 3 Department of Physiotherapy, Redeemer’s University, Ede, Osun State Nigeria; 4 Applied Health Sciences, Faculty of Graduate Studies, University of Manitoba, Winnipeg, Canada; 5 Department of Mental Health, College of Health Sciences, Obafemi Awolowo University, Ile– Ife, Nigeria; 6 Discipline of Physiotherapy, University of KwaZulu-Natal, Durban, South Africa; 7 Department of Physiotherapy, Nottinghamshire Healthcare NHS Foundation, Nottingham, UK; 8 School of Physiotherapy, Dalhousie University, Nova Scotia, Canada; 9 School of Social Work, McGill University, Montreal, Canada; 10 Medical Social Services Department, University College Hospital, Ibadan, Nigeria; 11 Department of Nursing Education, University of the Witwatersrand, Johannesburg, South Africa; 12 Department of Health Professions, Faculty of Health, Psychology and Social Care, Manchester Metropolitan University, Manchester, Great Britain

**Keywords:** Social support interventions, Depression, Quality of life, Older adults

## Abstract

**Background:**

Interventions that include participation in social or group connections as measures to prevent or reduce depression have received little attention. This systematic review and meta-analysis aimed to determine the effects of social support interventions on depressive symptoms and Quality of life (QoL) among older adults.

**Methods:**

A detailed search of six databases comprising Medline, CINAHL, PubMed, Cochrane Library, African Journals Online and Web of Science Core Collections was conducted until January 2025. A review protocol was developed and registered with the PROSPERO database (ID-CRD42021283342). A meta-analysis was used to synthesize the findings of the included studies based on subgroups of social support interventions. Of the 1524 articles found from the six databases, only 16 randomised controlled trials (14 parallel and 2 cluster) were eligible for inclusion.

**Conclusion:**

Social support interventions included emotional support, social engagement, instrumental, instrumental and appraisal, and social engagement and appraisal support. Meta-analysis findings indicated that social support interventions had non-significant effects on depression and QoL among older adults. Social support interventions have the potential to reduce depressive symptoms and improve QoL. However, current evidence is insufficient to determine the impact of social support interventions on depression and QoL in older adults.

**Supplementary Information:**

The online version contains supplementary material available at 10.1186/s12877-025-06146-7.

## Background

There is an anticipation that the older adult population (*≥* 60 years) would triple by 2050 from 524 million of 2010, representing 16% of the world’s population [1, 2]. This tripling in older adult population is likely to escalate the prevalence of chronic illnesses (such as heart diseases, cancers, and chronic respiratory diseases), psychological impairments (i.e., depressive symptoms and cognitive decline), and poor Quality of life (QoL) associated with ageing [3–5].

Depression is a major psychological impairment in the older adult population characterised by symptoms including long-lasting feelings of sadness, weight loss, and lack of interest in enjoyable activities [6]. Depression frequently increases with age [7] affecting approximately 1–5% of the older adult population [8]. Depression is not only a barometer of psychosocial well-being, but also a significant predictor of life expectancy [9, 10]. Further, depression is associated with premature mortality, significant disability and a decline in cognitive function [11, 12]. Depression, occurring alone or in the background of other chronic illnesses, may lead to worsened health outcomes and poor QoL among older adults. Age-related decline in social activities is linked to poor psychosocial health in older adults and ranked high among the risk factors for depression and decreased QoL [13, 14].

Conceptual models of social support have opined that social support may be instrumental to the ability of individuals at coping with stress and depression while also improving their mood, and general well-being [15, 16]. Scholars have argued that better social support and engagement in social activities lowers the risk of cognitive decline, mortality, and the propensity for hospitalisations [17–20]. In addition, social support improves self-efficacy [21, 22] and decreases depression among older adults [23, 24]. The scope of social support is broad and is typically measured in several ways: the structure (network size); the functions (instrumental, appraisal, informational and emotional) of social networks; enacted support (the actual provision of support), and the recipient’s subjective experience of support [25]. For instance, laughter therapy, an emotional support intervention, involves activities and exercises that encourage laughter and reminiscences, fostering a sense of connection and belonging [26]. This approach helps to alleviate depressive symptoms in older adults, ultimately improving their Quality of life [27]. Additionally, other social supports such as instrumental and appraisal support with self-management or financial assistance for medications, access to relevant health information, and affirmation can provide practical help for daily living [28]. Social engagements such as participation in senior centre activities and gerotranscendence support can also help older adults maintain connections and engage with their community [29]. Interventions that include participation in social or group connections as measures to prevent or reduce depression have received little attention [30, 31]. Further, systematic reviews have explored the effectiveness of physical activity interventions on healthy ageing [32–35], leaving out the impact of social interaction on health outcomes of the older adults. Thus, this systematic review and meta-analysis aimed to summarise evidence on the effects of interventions involving social support on depressive symptoms (the primary outcome for this review) and QoL (a secondary outcome) among older adults.

## Methods

### Design

This review was conducted following the preferred reporting items for systematic reviews and meta-analysis (PRISMA) guidelines [36]. A review protocol was developed and registered with the PROSPERO database (International Prospective Register for Systematic Reviews, CRD42021283342).

### Search strategy

Search for articles was conducted using six electronic databases: African Journal Online (AJOL), MEDLINE (via OVID), Cumulative index to nursing and Allied Health Literature (via EBSCOhost), Web of Science Core Collections, PubMed Central and Cochrane Library (from inception to March 2023 and updated 21 st January 2025). The search strategy was reviewed by a team of experts from different health professions and an experienced researcher in systematic review methodology. The following search terms, in combination with keyword and medical headings, were used and modified in each database (Table S1) (effect OR effectiveness) AND (‘social support’ OR ‘social interaction’ OR ‘peer support intervention’ OR ‘social engagement’ OR ‘family network’) AND (‘older adults’ OR ‘elderly’ OR ‘senior citizens’ OR ‘retirees’ OR ‘retired people’ OR ‘old people’) AND (‘depression’ OR ‘depressive symptoms’ OR ‘depressive mood’) AND (‘quality of life’ OR ‘well-being’ OR ‘health-related quality of life’). Further, the reference lists and citations of eligible full text articles were checked for more eligible articles.

### Study criteria and selection

#### Population, intervention, comparators, outcomes, study types and settings

The population of interest of this review was older adults aged 60 years and above, or a mixed population reporting subgroup analysis on older adults aged 60 and above. The intervention is a social support intervention, which we operationalised to encompass emotional support, instrumental support, appraisal support and social engagement (Table S2). We included all types of randomised controlled trials from all settings. The comparators included standard care, placebo, usual care or no intervention. The primary outcome of the review was depression using a self-reported instrument, while the secondary outcome was QoL, which is a composite of physical, psychological and social domains of perceived health.

#### Inclusion and exclusion criteria

Studies were included if they met the following criteria: (a) study population of older adults (≥ 60 years), or a mixed population reporting subgroup analysis on older adults; (b) randomised controlled trials (RCTs) that explored the effects of interventions involving social support on self-reported depressive symptoms and/or QoL among older adults; (c) published in English. Studies having participants with dementia, cognitive impairment, and neurocognitive and neurodegenerative disorders and animal studies were excluded. In addition, non-English language articles or grey literature were excluded.

### Study selection

Two members of the review team (BE and HF), independently screened identified studies for titles and abstracts. Thereafter, the two reviewers also independently screened eligible full texts against the inclusion criteria. In cases of disagreement at each stage, the two reviewers discussed and reached a consensus. A third member of the review team (OI) was consulted for final decision where the two reviewers could not reach consensus.

### Data extraction

Three reviewers (BE, TA and MI) independently extracted data from the included studies using a pre-tested standardised data extraction Excel spreadsheet. The CONSORT [37] and TIDieR [38] frameworks were used to guide the report of the intervention components and comparators. From each article, sample details, study design, intervention description, outcomes used to assess depressive symptoms, QoL, and the main findings of each study were extracted. Two reviewers (HF or OI) reconciled conflicts in the data extractions when the three reviewers were unable to reach a consensus after discussion. Data extraction was performed using Microsoft Excel 2016 (version 15. Microsoft Corporation, Redmond, WA, USA).

### Quality appraisal

The methodological Quality of the included articles was independently appraised by three assessors (BE, TA and MI) using the revised Cochrane risk of bias tool for randomised controlled trials (RoB-2). The three assessors pilot tested the risk of bias tool to familiarise themselves with the tool and to ascertain consistency. If the three assessors could not reach a consensus after discussion in cases of discrepancies, HF or OI were consulted as necessary. Irrespective of the methodological Quality or risk of bias, we included all studies in this review.

### Data synthesis

Meta-analysis was performed using Review Manager (RevMan Version 5.4.1). Social support intervention was operationalised to encompass emotional support, instrumental support, appraisal support and social engagement (Supplementary Material S2). A critical look at the interventions was considered, we were unable to pool all the interventions together based on the different types of social support interventions. Hence, we did a subgroup meta-analysis by social support intervention types. In cases where two social support interventions were combined, we pooled these data together and did a sub-group analysis. We used a standard mean difference (SMD) with 95% confidence interval (95% CI) to summarize effect estimates for depression and QoL. Random effect model was used based on the assumption that the effect estimates of different studies varied and distributed around an average of the effects [39]. We quantified statistical heterogeneity using the I^2^ statistic, an estimation of the variability in the effect estimates of the different studies [40].

## Results

### Search results

**Fig. 1:**
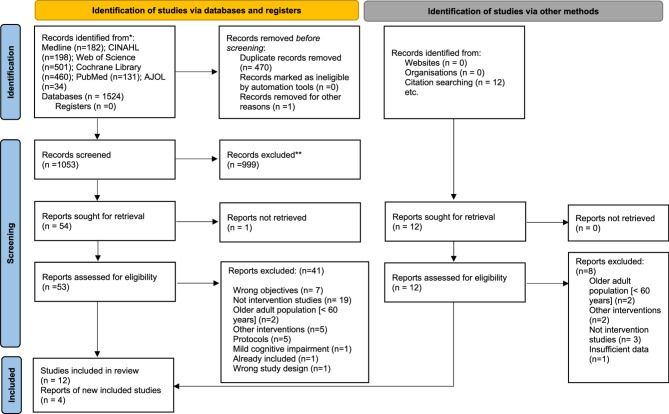
PRISMA flow diagram of the study selection

We identified 1524 articles in the electronic databases, and these citations were exported to RAYYAN– Al powered tool for systematic literature review. After removing duplicates and screening for titles abstracts and full texts, twelve studies were found eligible for inclusion; reference list and citation searching of these eligible studies yielded an additional four articles. The 16 articles included in the final review were [26, 27, 41–54]. The PRISMA flow chart of study selection is presented in Fig. 1.

### Study characteristics

Fourteen of the 16 included studies were parallel randomised controlled trials (RCTs) designs [26, 27, 41, 42, 44–47, 49–53] while two were cluster RCT designs [43, 54]. The studies were conducted in eleven countries: China [52], Norway [41], Taiwan [44, 53], USA [45, 46], Iran [27], Japan [47, 50], Hong Kong [48], Canada [49, 54], Turkey [26], Israel [51], Spain [42]. Fourteen studies (parallel RCTs) out of the 16 included had 2,168 participants [26, 27, 41, 42, 44–48, 50–53] while the two cluster RCT studies had 157 participants [43, 54]. Sample sizes for the included studies ranged from 36 [42] to 390 participants [46]. Majority of the studies included both genders but had a preponderance of female than male participants, although, Chiang et al. included only male participants [44] and Shapira et al. did not specify the sex of participants included [51]. 56% of studies were community-based [27, 43, 45–50, 52] while the other studies were conducted in a senior centre [41], a retirement home [42], nursing homes [26, 44] and online [51, 53, 54]. Table 1 shows the summary of articles included in this review with study design, sample details, interventions description, outcomes used to assess depression and QoL, as well as the study’s main findings.

**Table 1:** Characteristics of the included studies

Authors (year); country/type of study design	Operationalisation of social support (SS)	Total sample size; sex(m/f); dropout Age: mean (sd)/range	Description of social support intervention	Intervention received by control group	Mode of delivery; duration, frequency, intervention provider/settings	Outcome measures for Depressive symptoms/Quality of life (QoL)	Main findings	Implications/Limitations
Bøen et al., (2012) [41] Norway/Parallel RCT	SS was operationalised as social engagement by participating in common senior centre activities in Oslo, Norway	Total sample *n* = 138; IG = 77, CG = 61; sex m/f = 59/79; dropout = IG = 40, CG = 6 Mean age = not reported Age range: 65–80+	Each group had a certain number of members, ranging from 7 to 10. A self-help group discussed themes that the participants choose themselves, such as home and outdoor safety, how to avoid falling, social relationships and aging, and humour and laughing.	The control group was free to continue daily activities as they choose. They were administered same group activities as the intervention group after 1 year without a follow up.	Group of 7–10 participants, 35–38 sessions in a year, in three senior centres delivered by physiotherapists, nurses and senior centre leaders Community	**Depressive symptoms**: Beck Depression Inventory (BDI) **QoL**: Life satisfaction questionnaire (LSQ) was measured on scores on a question about QoL	**BDI**: IG: Baseline = 10.14 (6.63), post = 10.70 (5.95) at 12months $\:\varDelta\:=$0.56 (5.45) CG: Baseline = 8.7 (4.85), post = 9.44 (4.19) at 12months $\:\Delta\:=$0.74 (4.72) Effect size = Cohen’s D = 0.03 **LSQ**: IG: Baseline = 3.65 (0.82), post = 3.59 (0.76) at 12months $\:\varDelta\:=\:$- 0.06 (0.78) CG: Baseline = 3.84 (0.71), post = 3.61 (0.79) at 12months$\:\Delta\:=$− 0.22 (0.74) Effect size = Cohen’s D = 0.22	**IMPLICATIONS** Based on current findings of the lack of massive significance on effect size, this intervention cannot yet be recommended for treatment. **LIMITATIONS** The high percentage of dropouts, which may have resulted in a selection bias by making it difficult to conduct a truly fair comparison across the group, is a major limitation. And not finding the intention-to-treat analysis is another limitation
Chen et al., (2019) [43] Taiwan/Pragmatic cluster RCT	SS was operationalised as social engagement by participating in multidimensional support program (MSP)	Total sample *n* = 98; IG = 49, CG = 49; sex (m/f) = 22/76; dropout = not reported Mean age= IG = 73.8 (5.9) CG = 73.3 (5.6) Age range: IG = 65–88 CG = 65–87	The multidimensional support program (MSP) was a modified program based on a gerotranscendence support group that included topics such as recognizing gerotranscendence, typical aging symptoms and processes, limitations, and adjusting to the aging process. The MSP had three stages: 30-minutes of knowledge education, 15 min of interaction activity, and 15 min of sharing and discussing experiences.	During the intervention period, the control group got usual care such as fitness, art, and relaxation activities such as painting and singing, but no information about gerotranscendence was provided.	A large group comprising of 8 sessions, each lasted 60 min per week for 8 weeks and had 3 stages, was delivered by psychiatrist, psychologist, social worker, mental health nurse and community health nurse. Community	**Depressive symptoms**: The 15-item Geriatric Depression Scale-Short Form (15-item GDS-SF) **QoL**: Nil	**GDS-SF**: IG: Baseline = not clear, post = 2.2 (1.5) at 8 weeks $\:\varDelta\:=$not reported Effect size = not report CG: Baseline = not clear, post = 2.5 (1.8) at 8 weeks, $\:\Delta\:=$not reported Effect size = not reported	**IMPLICATIONS** MSP is a potential health promotion program which can be used as part of health promotion program for older adults to improve their mental and spiritual health. However, more studies are needed to confirm its usefulness. **LIMITATIONS** The limitations of the study include low scores of the GDS-SF. Low Cronbach’s alpha for the Chinese version of the gerotranscendence indicating issues with validity of gerotranscendence in Taiwanese population.
Gustafson et al., (2021) [46] USA/Parallel RCT	SS was operationalised as instrumental support by participating in the available services on the ElderTree website	Total sample *n* = 390, sex (m/f) = 98/292; IG = 197, CG = 192 drop out = IG = 38, CG = 41 Mean age = 76.5 (7.4) Age range: not reported	A few services offered by ElderTree intervention website included thoughts of the day, search, new content alerts, my to-do list, my health tracker, my bookmarks, my services, private messages, public discussions, family and friends, ask a coach, general resources, local resources, bulletin board, active living tips, map your trip, my profile, members and help. And usual sources of information and communication.	The control group received usual sources of information and communication.	12 months plus 6 months follow up Community	**Depressive symptoms**: 8-item Patient Health Questionnaire (PHQ) **QoL**: Patient-Reported Outcomes Measurement Information System (PROMIS)	**PHQ**: IG: Baseline = 0.71 (0.20), post = 0.72 (0.19) at 6 months; 0.72 (0.21) at 12 months $\:\varDelta\:=$0.01 CG: Baseline = 0.72 (0.20), post = 0.72 (0.19) at 6 months; 0.73 (0.22) at 12 months $\:\Delta\:=$0.01 Effect size = 0.04 (−0.18; 0.26) **PROMIS**: IG: Baseline = mental QoL = 3.40 (0.78); physical QoL = 3.42 (0.67), post = mental QoL = 3.42 (0.73) at 6 months; 3.45 (0.83) at 12 months; physical QoL = 3.42 (0.66) at 6 months; 3.42 (0.78) at 12 months $\:\varDelta\:=\text{m}$ental QoL = 0.05; physical QoL = 0.00 CG: Baseline = mental QoL = 3.32 (0.79); physical QoL = 3.42 (0.68), post = mental QoL = 3.36 (0.74) at 6 months; 3.40 (0.84) at 12 months; physical QoL = 3.44 (0.67) at 6 months; 3.46 (0.79) at 12 months $\:\Delta\:=$ental QoL = 0.08; physical QoL = 0.02 Effect size = mental QoL = 0.00 (−0.25; 0.25); physical QoL=−0.07 (−0.32; 0.18)	**IMPLICATIONS** ElderTree as an instrumental social support intervention is effective in the management of QoL and depression among older adults. **LIMITATIONS** Participants were not blinded to the condition once they were randomized: memory bias may affect self-reports.
Heidari et al., (2020) [27] Iran/Parallel RCT	SS was operationalised as social engagement support by participating in leisure-fun activities (laughter therapy)	Total sample *n* = 90; sex (m/f) = 28/62; dropout = 0 Mean age = not reported Age range: 60–69	Each session’s intervention consisted of 30 min of playing musical and visual slides and humorous video clips, as well as 15 min of games with award for humor telling and joke telling (15 min). By participating in competitions and sharing jokes, patients actively and interactively participated in meetings.	The control group had no specific plan, but they were given the assurance that similar meetings as that of the intervention group would be held in the future in regards to ethics and to encourage cooperation with filling out the questionnaires.	10 sessions; 1 h/3 times per week Nursing homes	**Depressive symptoms**: Geriatric Depression Scale (GDS-15) **QoL**: Short Form-36 Quality of Life (SF-36 QoL)	**GDS-15**: IG: Baseline = 6.87 (3.62), post= 2.57 (2.35) study duration was not reported $\:\varDelta\:=$4.3 CG: Baseline = 5.7 (3.57), post= 6.02 (3.78) $\:\Delta\:=$ −0.32 Effect size = not reported **SF-36 QoL**: IG: Baseline = 47.15 (16.02), post = 59.96 (17.58) $\:\varDelta\:=$12.81 CG: Baseline = not reported, post = not reported $\:\Delta\:=$not reported Effect size = not reported	**IMPLICATIONS** The findings of this study indicate that laughter therapy is one of the low-cost, safe, and non-invasive therapies that reduce geriatric depression by boosting endorphin levels and elevating mood. Therefore, it is essential that this treatment program be employed to raise senior people’s Quality of life. **LIMITATIONS** The use of self-reporting tools, which is predicted on the possibility that subjects may not be totally honest when describing their issues and answering questionnaires. Additionally, because elderly people come from extremely various cultural, social, and familial backgrounds, there are restrictions on how conclusions, interpretations, and causes of variables under research can be generalized, which must be taken into account.
Imai et al., (2015) [47] Japan/Parallel RCT	SS was operationalised as instrumental support by receiving postcard from Kyoto one of the famous cultural centres in Japan.	Total sample *n* = 184; sex (m/f) = 53/131; dropout = 38, IG = 20, CG = 18 Mean age IG = 82.2(7.90), CG = 80.4(7.40)	For eight months, letters with pictures or illustrations were mailed in a sealed envelope and written on two pieces of A4 paper. The letter was divided into two sections: a handwritten note intended to foster social connections and computer-printed seasonal greetings or news of the month from Kyoto. The letter also included optional self-addressed, stamped reply letters. The return letters made it clear that a response was not required.	None	Intervention was delivered individually; once a month for eight months Community	**Depressive symptoms**: The 15-item Geriatric Depression Scale-Short Form (15-item GDS-SF) **QoL**: 100 mm visual analogue scale (VAS), Basic Activities of Daily Living (BADLs) and Advanced Activities of Daily Living (AADLs)	**15-item GDS-SF**: IG: Baseline = 8.2 (3.0), post= 7.7 (3.7) $\:\varDelta\:=$not reported CG: Baseline = 8.2 (2.8), post = 7.5 (3.5) $\:\Delta\:=$=not reported **100 mm VAS**,** BADLs**,** AADLs**: Results not clearly reported	**IMPLICATIONS** The use of postcard interventions to treat senior depression in rural Japan is neither practical nor efficient. **LIMITATIONS** Even though it was made clear that a response was not required, several participants felt burdened by this need, which may have something to do with Japanese culture. These participants were seniors. Some elderly people may have trouble seeing and writing.
Lai et al., (2019) [48] China/Parallel RCT	SS was operationalised as emotional support by participating in life story work program in Hong Kong	Total sample *n* = 244; sex (m/f) = 70/174; dropout, IG = 26, CG = 31 Mean age = 77.1 (not reported) Age range: 61–99	Participants were assisted in talking about their life stories using a semi-structured method at the first meeting, for example, about their birthplace, hometown and upbringing. Before moving on to discussing more about each participant, the volunteer briefly re-read the stories and validated the participants’ written content at the subsequent meeting. Other domains in a person’s life history were covered through numerous cycles of meetings and interviews (e.g., marriage and children). The participants’ sharing was facilitated by the usage of photos and souvenirs. A life storybook was the result of this biographical method.	The control group talked and focused on current events (such as discussing daily news and sharing recipes utilizing seasonal ingredients) and were discouraged from talking about their personal lives.	A one-to-one intervention; 4–6 sessions, 30–60 min weekly, 6 months. Community	**Depressive symptoms**: The 15-item Geriatric Depression Scale-Short Form (15-item GDS-SF) **QoL**: Nil	**15-item GDS-SF**: IG: Baseline = 3.61 (3.24) Post = 3.48 (3.21); at 3 months = 3.40 (2.90); at 6 months = 3.76 (3.06) $\:\varDelta\:=\:$not clear CG: Baseline = 2.95 (2.80) Post = 2.52 (2.75); at 3 months = 2.76 (2.32); at 6 months = 2.49 (2.62) $\:\Delta\:=$not clear Effect size = not reported	**IMPLICATIONS** Whilst, there was no statistically significant difference over time across the groups on depression. Nonetheless, some participants claimed that their life storybooks would enable them to share more personal information with their children and other people. **LIMITATIONS** The study was tightly controlled, but the intervention dose is definitely something that should be considered in future research. A fairly large numbers of participants were lost to follow up.
Lai et al., (2020) [49] Canada/Parallel RCT	SS was operationalised as emotional support by participating in peer support programme.	Total sample *n* = 60; sex(m/f) = 22/38; dropout = 0 Mean age = 80 (not reported) Age range: 61+	Each intervention group member was paired with two peer supporters throughout the first week. Over the coming weeks, every member of the intervention group received two-on-one peer support services through home visits, phone calls, and other activities like emotional support, referrals, assistance with setting goals like self-care and social engagement, problem-solving, mental health and community resources. Because only one staff member or volunteer may be sent for a house visit per normal professional practice safety protocol, a two-on-one match was necessary. The volunteers attentively listened to and respected the requirements of the elderly participants despite not being able to offer professional assistance or therapy. Participants heard personal stories from peer supporters as well. Participants in the intervention group were invited to two monthly peer support group meetings led by a trained staff program coordinator with a background in social work and a registered social worker, where they met with other participants and peer supporters with the goal of fostering solid, sustaining relationships with other seniors.	The program coordinator only made brief calls to the individuals in the control group. The calls would not explicitly extend any invitation to actions that would trigger further engagement in social participation in programs or social activities, even though the call receivers might feel a sense of care, which is common in all forms of social interactions that the call receivers experienced in their routine daily interactions with others.	8 weeks Community	**Depressive symptoms**: General Depression Scale-4 (GDS-4) **QoL**: Nil	**GDS-4**: IG: Baseline = *n* = 9 (30%), post = not reported $\:\varDelta\:=$7 (23.3%) (*p* = 0.0008) CG: Baseline = 6 (20%), post = not reported $\:\Delta\:=$ −1 (3.33%) Effect size = not reported	**IMPLICATIONS** The program was not effective in improving depression among the participants. **LIMITATIONS** Small sample size. Whilst using this strategy would be morally acceptable, it also explains why the intervention group participant’s mean age is higher than that of the control group, thereby jeopardizing the validity of the study’s findings. Thirdly, there was only one post-test in this study and no other follow ups.
Saito et al., (2012) [50] Japan/Parallel RCT	SS was operationalised as emotional support by participating in a program designed for elderly migrants	Total sample *n* = 63; sex (m/f) = 20/43; dropout = 3 (IG = 1, CG = 2) Mean age= IG = 72.6(4.4), CG = 72.8(4.8); Age range: 66–84	The first session gave participants an overview of the intervention programs’ content, gave them a chance to get to know the elderly migrants and program staff members, and gave them some background knowledge of city A. The second session was dedicated to a focus group discussion on how participants’ relocation experiences had affected their daily lives.	People in this group had to wait for 7 months before participating in the program. Various newsletters or written information about group activities of city A were sent during the intervention period.	4 sessions 2 h each, once in 2 weeks for 6 months Community	**Depressive symptoms**: The 15-item Geriatric Depression Scale-Short Form (15-item GDS-SF) **QoL**: Nil	**15-item GDS-SF**: IG: Baseline = 4.6+_3.5, post = not reported $\:\varDelta\:=$not reported CG: Baseline = 5.0+_3.2, post = not reported $\:\Delta\:=$not reported Effect size = not reported	**IMPLICATIONS** The program did not significantly improve depression **LIMITATIONS** The study’s sample size was small and was not blinded.
Conwell et al., (2020) [45] USA/Parallel RCT	SS was operationalised as instrumental support (social connection) by participating in The Senior Connection Program in New York, USA	Total sample *n* = 369; sex (m/f) = 166/203; dropouts, IG = 34, CG = 22 Mean age = 71 (not reported) Age range: 60–97	Peer companions were told to make friendly visits rather than perform tasks that were purely instrumental and had no social value, like housework. At least two in-persons visits were required of the four prescribed contacts per month; the remaining interactions might be phone calls.	The comparison group required no participation in Lifespan’s peer companionship program during the follow up period. Other informal social assistance or social services initiatives, as well as medical or psychiatric therapies, were not prohibited.	An average of one in-person meeting lasting 1.75 h (range 0–5 meetings; 0–19 h of visiting time) per month, and two calls lasting an average of 31 min in total (range 0–6 calls; 0–35 h). Home setting and home call	**Depressive symptoms**: Patient Health Questionnaire-9 item (PHQ-9) **QoL**: Nil	**PHQ-9**: IG: Baseline = 8.67 (0.91), post= 6.34 (0.90) at 12months $\:\varDelta\:=$ −2.33 CG: Baseline = 8.28 (0.91), post= 6.96 (0.94) at 12months $\:\Delta\:=$ −1.32 Effect size = not reported	**IMPLICATIONS** The intervention improved depression among the participants. **LIMITATIONS** Researchers and carers could not realistically be blinded to condition, which limited the study.
Chiang et al., (2009) [44] Taiwan/Parallel RCT	SS was operationalised as emotional support by participating in reminiscence therapy in Taipei, Taiwan	Total sample *n* = 130; sex (m/f) = all males; dropout = 38 (IG = 20, CG = 18) Mean age = 77.24 (3.97) Age range: not reported	The sessions were organized and focused on a different subject every week. Recalling family history and life stories, transitional issues, becoming aware of one’s own accomplishments and identifying personal goals, identifying positive strengths, and increasing participants’ awareness of their feelings were some of the therapy topics covered.	To guarantee identical administration practices for all groups, research adhered to written guidelines. After this study was over, the waiting list control group’s participants engaged in additional reminiscence therapy.	8 sessions; 90 min, once per week for 8 weeks in a nursing home institution in the Taipei area delivered by mental health nursing student. Nursing home	**Depressive symptoms**: Center for Epidemiological Studies Depression scale (CES-D) **QoL**: Nil	**CES-D**: IG: Baseline = 19.11 (2.12), post = 16.18 (2.07); follow up = 15.49 (1.99) at 3 month $\:\varDelta\:=$*p*= −2.93, f=−3.62 CG: Baseline = 18.91 (2.98), post = 18.74 (2.70); follow up = 19.43 (2.22) at 3 months $\:\Delta\:=$*p*= −0.17, f = 0.52 Effect size = not reported	**IMPLICATIONS** The program improved depression among the participants in the IG post intervention and follow up in relative to the control group. **LIMITATIONS** The study’s limitations included the fact that it was almost entirely restricted to elderly people (males) from a single institution. The subjects’ comprehension of the study’s information was constrained (*n* = 51 high level of illiteracy) and that there was a dropout rate of about 30%.
Wong et al., (2022) [52] China/3-armed RCTs	SS was operationalised as instrumental support and appraisal support by participating in mHealth intervention.	Total sample *n* = 221; sex(m/f) = 36/185; dropout = 0 Mean age = 76.56(7.96) Age range: 60–98	mHealth group and mHealth + activity. Trained staff helped participants to download and use the mHealth app. The app has several features, including monitoring of vital signs, scheduling appointments, notification of medications and the dissemination of updated health education. A nurse monitored the vitals of the participants daily in the app database and when abnormalities were found, she called and assessed the participant within 24 h via a smartphone and follow up a working protocol to either educate the participants in self-care techniques and knowledge or refer the participants to a hospital. There was also a button installed in the app for participants to call a nurse when need be. The participants were instructed to use the app daily for 3 months. And when they have not used for one week, a reminder message popped up on the screen of the smartphone. Participants in the mHealth + I group received 8 proactiive calls (first month: weekly calls, second/third months: biweekly calls) from the nurse over the intervention period. This time the nurse did not only assess the physical health of the participants but their psychological health using a holistic tool, the Omaha system. Following assessment, the nurse provided psychological support by giving positive verbal encouragements during conversations.	The control group did not receive any mHealth or mHealth + I interventions.	Interventions were delivered by nurses for a duration of 3 months via smartphone apps and calls: first month: weekly calls, second/third months: biweekly calls. Community setting	**Depressive symptoms**: The Geriatric Depression Scale (GDS) **QoL**: 12-item Short Form Health survey version 2- Chinese version (SF-12 QoL)	**GDS**: IG: Baseline = mHealth group = 4.08(3.77), mHealth + 1 group = 4.47(3.59), post = mHealth group = 3.54(3.14), mHealth + 1 group = 3.81(2.84), follow-up at 3 months = mHealth group = 3.24(3.33), mHealth + 1 group = 4.41(3.46) $\:\varDelta\:=$mHealth group= −0.84(−0.44), mHealth + 1 group= −0.06(−0.13) CG: Baseline = 3.63(3.26), post= 3.75(3.08), follow-up at 3 months = 3.92(3.38) $\:\Delta\:=$0.12(−0.18) Effect size = not reported **SF-12 QoL**: IG: Baseline = mHealth group = 52.31(10.94), mHealth + 1 group = 49.71(10.80), post= mHealth group = 50.40(10.15), mHealth + 1 group = 49.51(9.43), follow-up at 3 months = mHealth group = 51.79(10.69), mHealth + 1 group = 47.92(10.39) $\:\varDelta\:=$mHealth group= −0.52(−0.25), mHealth + 1 group= −1.79(−0.41) CG: Baseline = 50.58(11.56), post = 49.67(11.01), follow-up at 3 months = 49.50(12.67) $\:\Delta\:=$ −1.08(1.11) Effect size = not reported	**IMPLICATIONS** Mhealth app intervention significantly improved depression and Quality of life of the community dwelling older adults. **LIMITATIONS** The study was limited to only older adults who had smartphones and internet coverage at home. Further, there was limited time to record long term effect of the intervention.
Yang et al., (2022) Taiwan/Parallel RCT	SS was operationalised as instrumental support by participating in online interactive courses.	Total sample *n* = 89; sex(m/f) = 32/57; dropout = 0 Mean age= 68.07(6.68) Age range: not reported	A group intervention was made accessible to participants through social network application called LINE from 12:00 to 20:30 which they could access via smartphones and computers. Each of the day course started with noon greeting (online interaction with LINE editor) followed by online interactive classes (1 h) and selected news and youtube vidoes in the afternoon, and concluded with music broadcast and evening greetings. Additionally, at the end of the course activity of the day the participants would be asked to take photos or send a sticker to indicate completion of the activity and ensure the Quality of the activity.	The control group joined a group in the social networking app (LINE) where the LINE editor sent only messages such as morning greetings, news and youtube videos without delivering an online interactive course.	Intervention was delivered by LINE editor for a duration of 8 weeks, 5 days in a week, 8 h. Online	**Depressive symptoms**: The Geriatric Depression Scale (GDS) **QoL**: WHOQOL-BREF	GDS: IG: Baseline = 1.61(1.45), post= 1.45(1.53) $\:\varDelta\:=$ −0.16(0.08) CG: Baseline = 1.62(1.92), post= 1.49(1.83) $\:\Delta\:=$ −0.13(−0.09) Effect size = − 0.28 **WHOQOL-BREF**: IG: Baseline = physical health = 15.47(2.32), psychological health = 14.80(2.35), social relationships = 14.14(1.79), environment = 15.54(2.36), post= physical health = 15.71(2.07), psychological health = 15.51(1.82), social relationships = 15.38(2.02), environment = 15.97(1.73),$\:\varDelta\:=$physical health = 0.24(−0.25), psychological health = 0.71(−0.53), social relationships = 1.24(0.23), environment = 0.43(−0.63). CG: Baseline = physical health = 15.80(2.27), psychological health = 15.56(2.53), social relationships = 14.83(2.57), environment = 16.01(2.36), post= physical health = 15.82(2.19), psychological health = 15.28(2.42), social relationships = 15.05(2.52), environment = 16.00(2.68), $\:\Delta\:=$physical health = 0.02(−0.08), psychological health = minus 0.28(−0.11), social relationships = 0.22(−0.05), environment = minus 0.01(0.32). Effect size = physical health = 0.15, psychological health = 0.64, social relationships = 0.50, environment = 0.28	**IMPLICATIONS** This study showed that 8-week intensive online interactive course effectively improved domains of psychological health and social relationships of the QoL of the older adults during the COVID-19 pandemic. However, the intervention had no significant effect on depressive symptoms. **LIMITATIONS** Exclusion of older adults who could not use smartphones limits generalisability of findings.
Alici & Bahceli, (2020) Turkey/Parallel RCT	SS was operationalised as social engagement support by participating in leisure-fun activities (Laughter therapy)	Total sample *n* = 68; sex (m/f) = 32/30; dropout = 6 (IG = 3, CG = 3) Mean age= IG = 72.4, CG = 73 Age range: not reported	Laughter therapy was administered to participants twice weekly for 6 weeks between 10am– 12 noon after breakfast. The intervention comprised warm up (gentle stretches and hand clapping (10 min)]; deep breathing exercises and hand clapping (5 min); children’s games and laughter exercises (15 min); and breathing exercises and meditation (10 min)	Usual care comprising routine nursing care and access to geriatric consultation. Participants also continued with their daily routine	Intervention was delivered to participants in a large group by a certified yoga laughter instructor twice a week for 6 weeks Nursing home	**Depressive symptoms**: Nil **QoL**: 5 Item Satisfaction with Life Scale (SLS)	**SLS**: IG: Baseline = 11.7 (3.21), post = 11.67 (4.19) at 6 weeks,$\:\varDelta\:=$not reported Effect size = 0.006 CG: Baseline = 10.77 (4.14), post = 9.63 (3.03) at 6 weeks,$\:\Delta\:=$not reported Effect size = 0.006	**IMPLICATIONS** Life satisfaction did not improve following laughter therapy. Cultural differences and demographic factors may influence findings. **LIMITATIONS** The study was carried out only in one institution.
Shapira et al., (2021) [51] Israel/Parallel RCT	SS was operationalised as social engagement by providing a safe place for social interaction	Total sample *n* = 86; IG = 64, CG = 18; sex (m/f) = not specified; dropout IG = 13 (17%) Mean age: 72 (5.63) IG = 72.1 (5.3) CG = 71.7 (6.8) Age range: 65–90	The aim of the intervention was to provide a place for social interaction and to create a learning space for coping skill required during the period of the study (COVID 19 pandemic). Each session lasted 60–90 min and comprised: (a) a guided group discussion (20–30 min), (b) learning and practice of cognitive behavioural skills and techniques such as relaxation, guided imagery of “a safe place”, identifying non-adaptive cognitive schemas, cognitive restructuring, constructive positive self-talk, and mindfulness (40–60 min)	Wait list control	Small group (5–7) guided sessions via zoom, 7 sessions, twice weekly, moderators were clinical social workers trained by one of the researchers, a clinical social worker. Online	**Depressive symptoms**: Nine item depression severity measure of the Patient Health Questionnaire (PHQ-9) **QoL**: **Nil**	**PHQ-9**: IG: Baseline = 6.3 (5.3), post = 5.2 (4.7) at 3.5 weeks ∆=not reported Effect size = 0.21 CG: Baseline = 6.3 (5), post = 7.1 (6.1) at 3.5 weeks ∆=not reported Effect size= −0.13	**IMPLICATIONS** The study provides a potential intervention for improving mental health among older adults living alone or in remote places. However, well-powered RCTs are required to validate findings. **LIMITATIONS** Small sample size, unequal allocation to study arms, use of convenient sample.
Carcavilla-Gonzalez et al., (2024) Spain/Parallel RCT	SS was operationalised as social engagements, and appraisal supports by participating in person centred support sessions	Total sample *n* = 36; sex (m/f) = 8/12; dropout = 16 (IG = 8, CG = 8) Mean age = not reported Age range: 65–99	Intervention comprised a 60-minute individualized companionship visit tailored to participants unique biographies, preferences and needs. The individualized companionship was based on communication and social contact, an emotional component, and social interaction.	The control group received similar visits but were not tailored to their biographies, needs or preferences	Personalised counselling twice a week for three months delivered by three professional caregivers who were trained by a psychologist. Retirement home	**Depressive symptoms**: Yesavage Geriatric Depression Scale (GDS) **QoL**: EQ-5D Visual Analogue Scale	**Yesavage GDS**: IG: Baseline = 4.10 (2.64), post=(1.80 (1.31) $\:\varDelta\:=$not reported CG: Baseline = 2.00 (1.49), post = 2.7 (1.63), $\:\Delta\:=$not reported Effect size = not reported **EQ-5D** Visual Analogue Scale: IG: Baseline = 67.7 (16.92), post = 80.0 (1.54) $\:\varDelta\:=$not reported CG: Baseline = 86 (18.38), post = 73 (28.2) $\:\Delta\:=$not reported Effect size = not reported	**IMPLICATIONS** The intervention appears to be beneficial in reducing depression and improving Quality of life among older adults. However, the fact that there were baseline differences in these outcomes suggests bias. **LIMITATIONS** The study’s sample size was insufficient. The finding may not be generalisable because data was taken from one centre
Ysseldyk et., al., (2023) Canada/Cluster RCT	SS was operationalised as instrumental support and appraisal support by participating in technological training with or without connection with peers through Facebook.	Total sample *n* = 59; sex (m/f) = 19/40; dropouts = 17 Mean age = 85.44(5.99); Age range: 65–95	Technology training: participants were taught how to use Facebook to connect with friends and family over social media Technology + connection: In addition to being taught on how to use Facebook for social connection, a Facebook group was specifically created for participants to connect with each other over the social media for the first time.	Newspaper club control: Participants had a weekly in-person newspaper discussion group that was facilitated by one of the student researchers. The group was designed to mimic the technology training group., i.e., social face to face interaction without technology. Routine as usual control: Participants received no intervention; only participated in their day-to-day daily routine as usual.	Adults living in residential care were trained on how to use technology to connect to friends and family by a team of professional instructors in adult technology education. Online	**Depressive symptoms**: Eight item CES-D (CES-D-8) **QoL**: 5 Item Satisfaction with Life Scale (SLS-5)	**CES-D-8**: IG: Baseline = 13.11 (4.92), post=(12.98 (4.93) $\:\varDelta\:=$not reported CG: Baseline = 12.52 (3.94), post = 13.9 (3.86) $\:\Delta\:=$not reported Effect size = not reported **SLS-5** IG: Baseline = 19.13 (4.51), post = 20.64 (3.66) $\:\varDelta\:=$not reported CG: Baseline = 19.76 (3.61), post = 19.62 (3.84) $\:\Delta\:=$not reported Effect size = not reported	**IMPLICATIONS** The intervention did not significantly improve the depression and QoL compared with the control. However, this study is a feasibility study and robust RCTs are warranted. **LIMITATIONS** The sample size was small. There might have been some cofounding factors peculiar to each cluster that was not captured in the study analysis. More in-depth experience regarding the use of the technology by older adults were not captured.

*SS *Social support, *m *male, *f *female, *SD *Standard deviation, *N/R *Not reported, *IG *Intervention Group, *CG *Control Group, *QoL *Quality of Life, $\:\Delta\:$ change

### Social support interventions

The social support interventions reported in this review include emotional social support [44, 48–50], social engagement [26, 27, 41, 43, 51], instrumental [45–47, 53], instrumental and appraisal [52, 54], and social engagement and appraisal support [42]. These interventions were delivered by physiotherapists, psychologists, social workers, mental health nurses, community health nurses, yoga laughter instructor, adult technology educators and trained research assistants (Table 1).

### Risk of bias

**Fig. 2:**
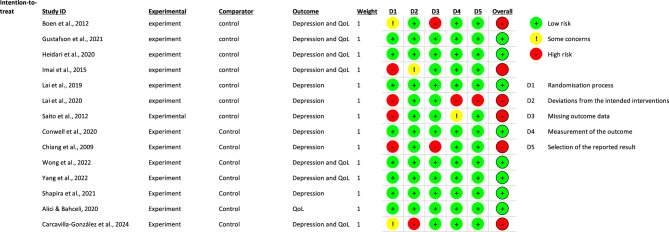
Cochrane risk of bias for parallel randomised controlled trials (RCTs)

**Fig. 3:**
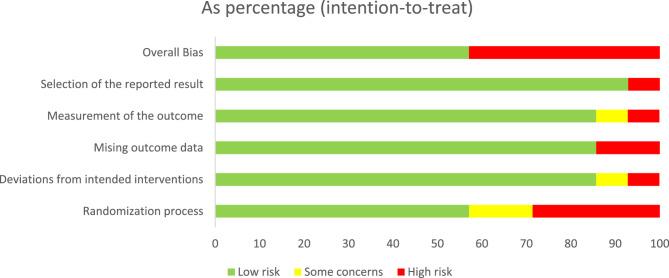
Risk of bias summary for parallel randomised controlled trials (RCTs)

**Fig. 4:**
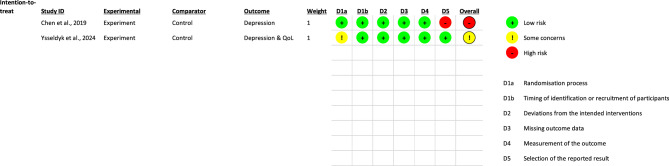
Cochrane risk of bias for cluster randomised controlled trials (RCTs)

**Fig. 5:**
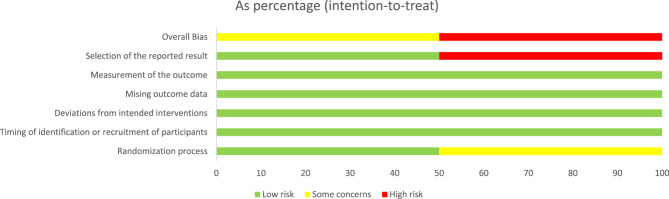
Risk of bias summary for cluster randomised controlled trials (RCTs)

From the fourteen parallel randomised trials, eight studies were rated to have a low risk of bias [26, 27, 45, 46, 48, 51–53] while six trials were adjudged to have a high risk of bias [41, 42, 44, 47, 49, 50] with the majority of the high-risk studies not reporting randomisation process details [44, 47, 49, 50]. The key reasons for the high risk of bias in trials included deviations from the intended intervention, missing outcome data, measurement of the outcome, and selective outcome reporting (Figs. 2 and 3). The two cluster trials were rated respectively as having high risk of bias due to selective outcome reporting [43] and having some concerns due to randomisation process [54] (Figs. 4 and 5).

### Meta-analysis of the intervention effects

#### Effects of social support interventions on depression and quality of life

**Fig. 6:**
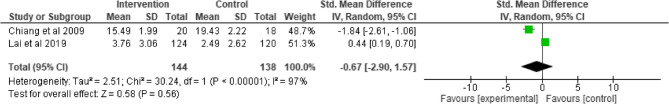
Emotional social support on depression

**Fig. 7:**
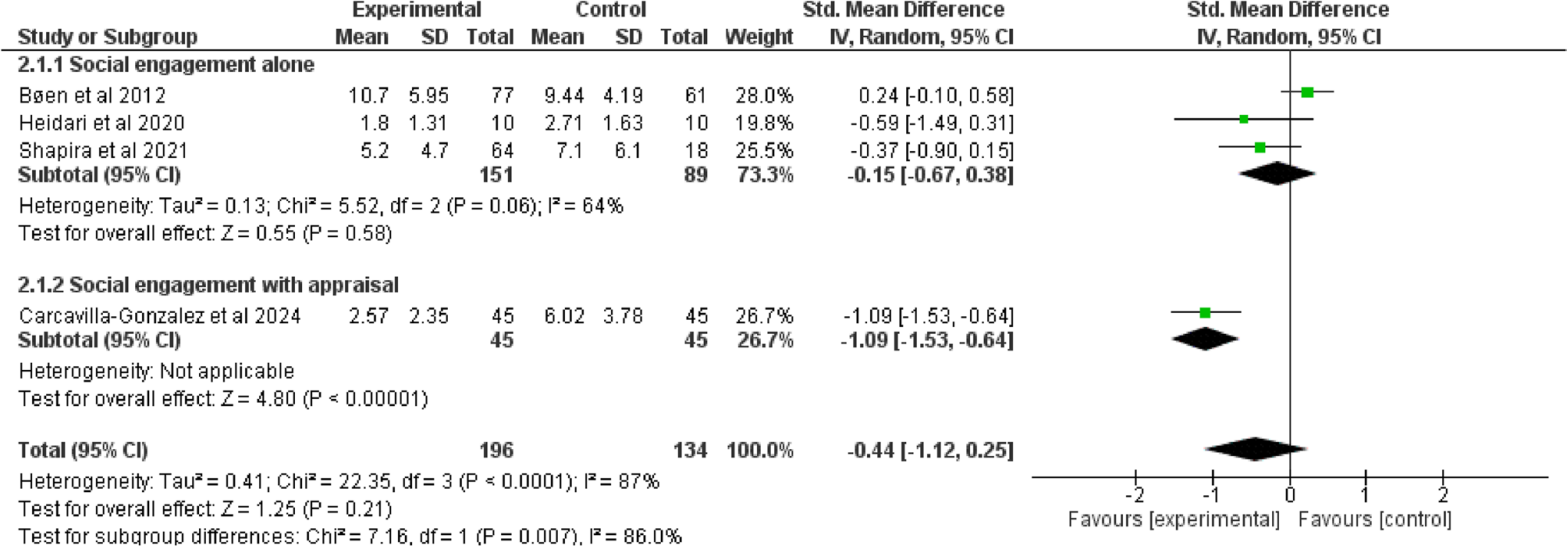
Social engagement without and with appraisal social support on depression

**Fig. 8:**
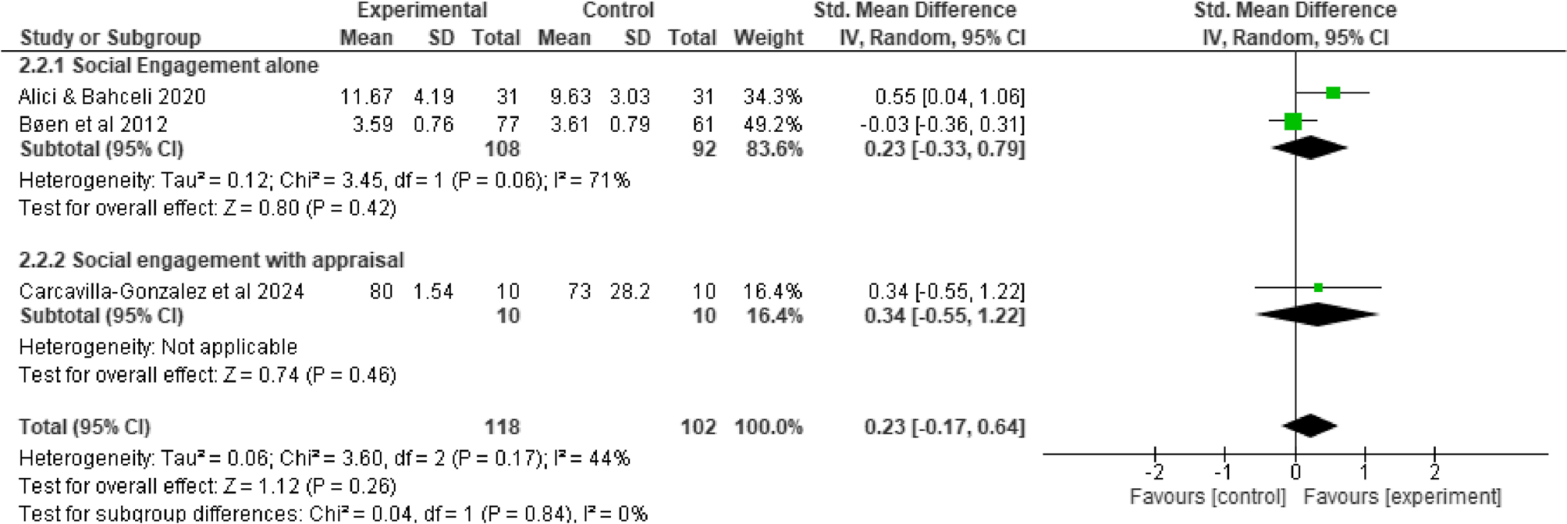
Social engagement with and without appraisal social support on Quality of life

**Fig. 9:**
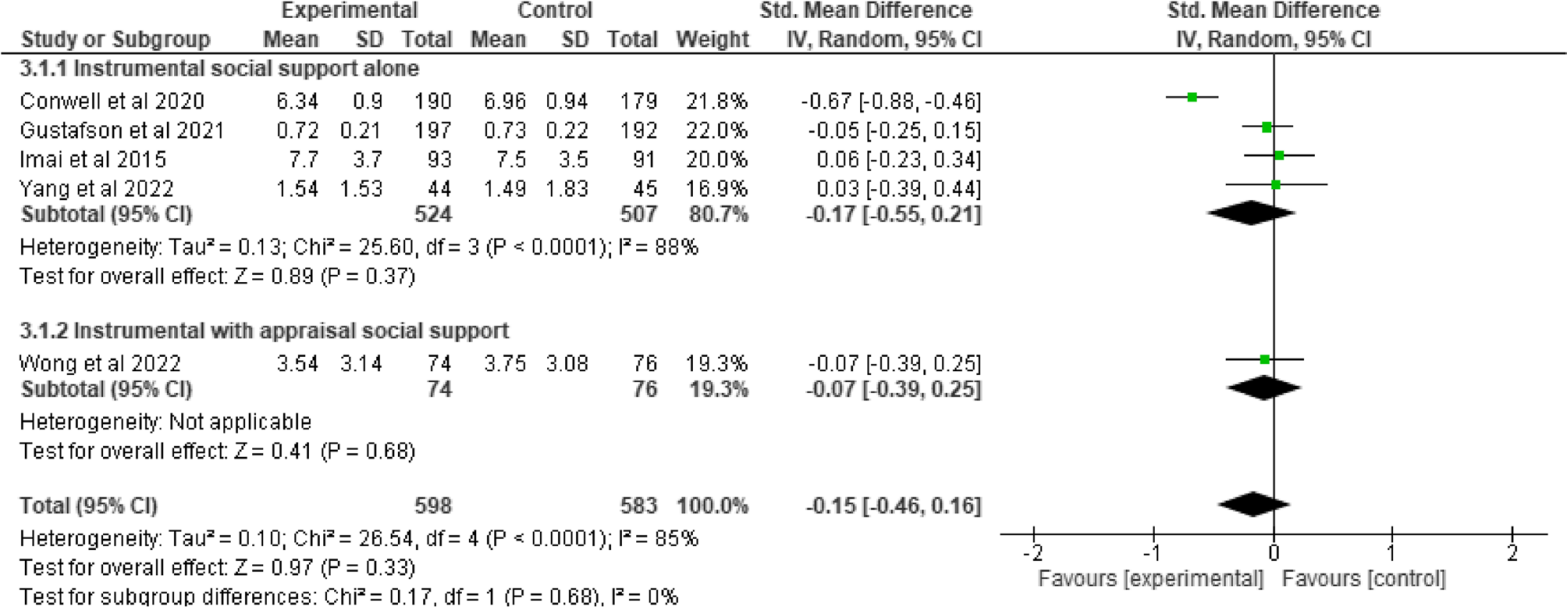
Instrumental social support with and without appraisal social support on depression

Compared to control interventions, emotional social support intervention did not lead to a significant improvement in depression among older adults (SMD = −0.67, 95% −2.90, 1.57; I^2^ = 97%, *p* = 0.56), (Fig. 6). Similarly, the effect of social engagement with and without appraisal social support intervention did not significantly improve depressive symptoms (SMD = −0.44, 95% −1.12, 0.25; I^2^ = 87%, *p* = 0.21) (Fig. 7) and Quality of life (SMD = 0.23, 95% −0.17, 0.64; I^2^ = 44%, *p* = 0.26), (Fig. 8). Furthermore, compared to control interventions, instrumental social support with and without appraisal social support had a non-significant reductive effect on depression among older adults (SMD = −0.15, 95% −0.46, 0.16; I^2^ = 85%, *p* = 0.33), (Fig. 9).

## Discussion

This systematic review and meta-analysis revealed that social support interventions had no significant effects on depression or Quality of life among older adults. While some individual programs showed promise, overall results were inconclusive. The synthesised evidence from 16 randomised controlled trials (RCTs) across diverse settings revealed that interventions were operationalised across four core domains of social support: emotional, instrumental, appraisal, and social engagement. The lack of significant effects may not reflect a failure of social support itself, but rather a misalignment between intervention models and the lived realities of ageing [13, 55]. Thus, the need for more rigorous studies that aligns with the cultural realities of older adults, and which in-turn improves their Quality of life.

Despite the global burden of depression among ageing populations and the growing advocacy for psychosocial interventions, these interventions are still at the infancy stage across many countries in the global south [56, 57]. This review revealed that social support interventions, whilst diverse and often culturally tailored, yielded limited statistically significant benefits on depression and QoL outcomes. These findings align with the broader notion of how most studies overlooked the relational nature of ageing and depression by reducing social support to discrete, time-bound programmes [58, 59]. Therefore, there is a potential promise in a contextually grounded, ethically attuned, and co-designed approaches that attend to the structural and emotional nuances of older adults’ lives.

Our review revealed that over 80% of eligible studies were situated in community-based settings, while social support interventions targeting older adults with depressive symptoms in geriatric centres and nursing homes remained significantly underrepresented [60]. This imbalance raises important concerns about how and where mental health interventions are prioritised across the spectrum of ageing care. The institutional invisibility of older adults in residential facilities—many of whom experience compounded vulnerabilities due to isolation, chronic illness, and restricted autonomy—speaks to a systemic neglect of structural and psychosocial determinants of mental health in these settings [13, 35]. Furthermore, the predominance of female participants in the reviewed studies aligns with epidemiological trends showing higher rates of depression in older women. However, it also underscores gendered patterns in help-seeking behaviour and research recruitment practices that often render older men invisible in mental health discourse [44, 61–63]. This gender imbalance limits the generalizability of findings and reinforces stereotypes that frame emotional vulnerability as a feminine trait, thereby stigmatising mental health concerns among older men. Future research must strive for a more gender-inclusive approach—one that both interrogates and resists normative assumptions around masculinity, ageing, and emotional well-being. Intentional efforts to destigmatise mental health and expand outreach among older men are vital to ensuring equity in both research and care [18, 57].

Despite the methodological limitations across studies, it is important to acknowledge the meaningful insights that emerged from some individual interventions—particularly those rooted in emotional connection and narrative, such as laughter therapy and life story work. These smaller-scale programmes, often delivered in intimate or culturally familiar settings, suggest that when older adults are met with interventions that affirm their identities, histories, and relational needs, there can be real improvements in mood and well-being [43, 44]. Yet, the overall evidence-base remains fragmented and inconclusive, largely due to a small number of studies and sample sizes, inconsistent outcome measures, and short follow-up periods. It is important to challenge the dominance of biomedical paradigms that frame older adults primarily through the lens of disease, decline, or productivity loss. Thus, there is an urgent need to reimagine psychosocial care for later life—not as an optional add-on, but as a core component of holistic, dignified aging. A sustained investment in rigorously designed, longitudinal research that honours the cultural, emotional, and social complexities of growing older across diverse communities is imminent. Only then can we begin to build interventions that are effective, just, inclusive, and truly responsive to the lived realities of ageing populations.

## Conclusion

From the findings of this review, social support interventions included social engagements, emotional, instrumental, and appraisal social supports. While the evidence remains inconclusive, glimpses of promise—particularly in culturally rooted, emotionally resonant approaches like laughter therapy and life story work—reveal the power of connection, narrative, and care. The dominance of community-based interventions also raises critical questions about who gets seen, supported, and prioritized in aging research and care. Older adults in institutional settings, and older men in particular, remain on the margins, pointing to the need for more inclusive, gender-sensitive, and context-specific research. As we move forward, it is essential to shift away from narrow biomedical models and toward holistic, human-centred approaches that honour the social, emotional, and cultural dimensions of ageing.

## Supplementary Information

Supplementary Material 1.

Supplementary Material 2.

## Data Availability

The datasets used and analyzed during the current study are available from the corresponding author upon reasonable request.

## Abbreviations

QoL: Quality of life
SMD: Standard mean difference
RCTs: Randomised controlled trials
CONSORT: CONsolidated Standards Of Reporting Trials
TIDieR: Template for Intervention Description and Replication
RoB-2: Risk of Bias Tool for Randomised Controlled Trials
PRISMA: Preferred Reporting Items for Systematic Reviews and Meta-Analysis
SID: Scientific information database
STROBE: Strengthening the Reporting of Observational Studies in Epidemiology for Cross- Sectional Study
