# Supplementary material for: Effects of social support interventions on depressive symptoms and quality of life among older adults: a systematic review and meta-analysis
Source: BMC Geriatr. 2025 Jul 9;25:512. doi: 10.1186/s12877-025-06146-7 (PMC12239414; doi:10.1186/s12877-025-06146-7)
Supplement: Supplementary file 1 — Supplementary Material 1. [file 12877_2025_6146_MOESM1_ESM.docx]

**Appendix 1**

Table S1

Search strategies for electronic databases (First Search – 23^rd^ and 24^th^ Feb., 2022 and updated 10^th^ March 2023)

| **Database** | **Search Terms** |
| --- | --- |
| Medline via Ovid  **n =146** | 1. (effect or effectiveness).mp. [mp=title, abstract, original title, name of substance word, subject heading word, floating sub-heading word, keyword heading word, organism supplementary concept word, protocol supplementary concept word, rare disease supplementary concept word, unique identifier, synonyms] = 3495614 2. limit 1 to (english language and humans) = 1852102 3. (social support or peer support intervention or social engagement or family network program or social interaction).mp. [mp=title, abstract, original title, name of substance word, subject heading word, floating sub-heading word, keyword heading word, organism supplementary concept word, protocol supplementary concept word, rare disease supplementary concept word, unique identifier, synonyms] = 105113 4. limit 3 to (english language and humans) = 94354 5. (older adults or elderly or senior citizens or retirees or retired people or old people).mp. [mp=title, abstract, original title, name of substance word, subject heading word, floating sub-heading word, keyword heading word, organism supplementary concept word, protocol supplementary concept word, rare disease supplementary concept word, unique identifier, synonyms] = 320984 6. limit 5 to (english language and humans) = 278306 7. (depression or depressive symptoms or depressive mood).mp. [mp=title, abstract, original title, name of substance word, subject heading word, floating sub-heading word, keyword heading word, organism supplementary concept word, protocol supplementary concept word, rare disease supplementary concept word, unique identifier, synonyms] = 407718 8. limit 7 to (english language and humans) = 307598 9. (quality of life or well-being or health related quality of life).mp. [mp=title, abstract, original title, name of substance word, subject heading word, floating sub-heading word, keyword heading word, organism supplementary concept word, protocol supplementary concept word, rare disease supplementary concept word, unique identifier, synonyms] = 405934 10. limit 9 to (english language and humans) = 360993 11. 2 and 4 and 6 and 8 and 10 = 146 |
|  |  |
| CINAHL (via EBSCOhost)  **n =158** | TI (effect OR effectiveness) AND (‘social support’ OR ‘peer support intervention’ OR ‘social engagement’ OR ‘family network program’ OR ‘social interaction’) AND (‘older adults’ OR ‘elderly’ OR ‘senior citizens’ OR ‘retirees’ OR ‘retired people’ OR ‘old people’) AND (‘depression’ OR ‘depressive symptoms’ OR ‘depressive mood’) AND (‘quality of life’ OR ‘well-being’ OR ‘health related quality of life’) = 1  AB (effect OR effectiveness) AND (‘social support’ OR ‘peer support intervention’ OR ‘social engagement’ OR ‘family network program’ OR ‘social interaction’) AND (‘older adults’ OR ‘elderly’ OR ‘senior citizens’ OR ‘retirees’ OR ‘retired people’ OR ‘old people’) AND (‘depression’ OR ‘depressive symptoms’ OR ‘depressive mood’) AND (‘quality of life’ OR ‘well-being’ OR ‘health related quality of life’) = 169  169 without limiters  157 with limiters – English language |
|  |  |
| Web of Science Core Collections  **n=363** | 1. (TI=(('effect' OR 'effectiveness'))) OR AB=(('effect' OR 'effectiveness')) = 12,979,611 2. (TI=('social support’ OR ‘peer support intervention’ OR ‘social engagement’ OR ‘family network program’ OR ‘social interaction')) OR AB=('social support’ OR ‘peer support intervention’ OR ‘social engagement’ OR ‘family network program’ OR ‘social interaction') = 411,738 3. (TI=(‘older adults’ OR ‘elderly’ OR ‘senior citizens’ OR ‘retirees’ OR ‘retired people’ OR ‘old people’)) OR AB=(‘older adults’ OR ‘elderly’ OR ‘senior citizens’ OR ‘retirees’ OR ‘retired people’ OR ‘old people’) = 643,391 4. (TI=((‘depression’ OR ‘depressive symptoms’ OR ‘depressive mood’))) OR AB=((‘depression’ OR ‘depressive symptoms’ OR ‘depressive mood’)) = 514,623 5. (TI=((‘quality of life’ OR ‘well-being’ OR ‘health related quality of life’))) OR AB=((‘quality of life’ OR ‘well-being’ OR ‘health related quality of life’)) = 608,298 6. #1 AND #2 AND #4 AND #5 AND #3 = 363 |
|  |  |
| Cochrane Library  **n=345** | (‘effect’ OR ‘effectiveness’) AND (‘social support’ OR ‘peer support intervention’ OR ‘social engagement’ OR ‘family network program’ OR ‘social interaction’) AND (‘older adults’ OR ‘elderly’ OR ‘senior citizens’ OR ‘retirees’ OR ‘retired people’ OR ‘old people’) AND (‘depression’ OR ‘depressive symptoms’ OR ‘depressive mood’) AND (‘quality of life’ OR ‘well-being’ OR ‘health related quality of life’) in Title Abstract Keyword - in Trials (Word variations have been searched) = 345 |
|  |  |
| AJOL  **n=23** | ‘effect’ OR ‘effectiveness’ AND 'social support’ OR ‘peer support intervention’ OR ‘social engagement’ OR ‘family network program’ OR ‘social interaction’ AND ‘older adults’ OR ‘elderly’ OR ‘senior citizens’ OR ‘retirees’ OR ‘retired people’ OR ‘old people’ AND ‘depression’ OR ‘depressive symptoms’ OR ‘depressive mood’ AND ‘quality of life’ OR ‘well-being’ OR ‘health related quality of life’ |
| PubMed Central  **n=87** | (‘effect’[Title/Abstract] OR ‘effectiveness’[Title/Abstract]) AND (‘social support’[Title/Abstract] OR ‘peer support intervention’[Title/Abstract] OR ‘social engagement’[Title/Abstract] OR ‘family network program’[Title/Abstract] OR ‘social interaction’[Title/Abstract]) AND (‘older adults’[Title/Abstract] OR ‘elderly’[Title/Abstract] OR ‘senior citizens’[Title/Abstract] OR ‘retirees’[Title/Abstract] OR ‘retired people’[Title/Abstract] OR ‘old people’[Title/Abstract]) AND (‘depression’[Title/Abstract] OR ‘depressive symptoms’[Title/Abstract] OR ‘depressive mood’[Title/Abstract]) AND (‘quality of life’[Title/Abstract] OR ‘well-being’[Title/Abstract] OR ‘health related quality of life’[Title/Abstract]) = 87 |

Table S2

| **Table S2: Operationalisation of Social Support** |
| --- |
| 1. Emotional support, indicating sharing life experiences, personal problems and caring (family networking). |
| 2. Instrumental (peer group counselling), like health Insurance, social network expansion, empowerment, self-determination and reduction of stigma. |
| 3. Appraisal support, involves positive feedback on the quality of one's performance thereby leading to an improved performance. |
| 4. Social engagement, social activities, championship and social participation in leisure and recreational activities, social networking. |
